# The implementation effectiveness of the ‘Strengthen your ankle’ smartphone application for the prevention of ankle sprains: design of a randomized controlled trial

**DOI:** 10.1186/1471-2474-15-2

**Published:** 2014-01-07

**Authors:** Miriam Van Reijen, Ingrid I Vriend, Victor Zuidema, Willem van Mechelen, Evert A Verhagen

**Affiliations:** 1 Department of Public & Occupational Health, EMGO + Institute for Health and Care Research and VU University Medical Center, Van der Boechorststraat 7, 1081 BT Amsterdam, The Netherlands; 2 Consumer Safety Institute VeiligheidNL, Amsterdam, the Netherlands

**Keywords:** Mobile health, Ankle sprains, Ankle injury, Prevention, Neuromuscular training

## Abstract

**Background:**

Ankle sprains continue to pose a significant burden to the individual athlete, as well as to society as a whole. However, despite ankle sprains being the single most common sports injury and despite an active approach by various Dutch organisations in implementing preventive measures, large-scale community uptake of these preventive measures, and thus actual prevention of ankle sprains, is lagging well behind. In an attempt to bridge this implementation gap, the Dutch Consumer Safety Institute VeiligheidNL developed a freely available interactive App (‘Strenghten your ankle’ translated in Dutch as: ‘**Versterk je enkel**; available for iOS and Android) that contains - next to general advice on bracing and taping - a proven cost-effective neuromuscular program. The ‘Strengthen your ankle’ App has not been evaluated against the ‘regular’ prevention approach in which the neuromuscular program is advocated through written material. The aim of the current project is to evaluate the implementation value of the ‘Strengthen your ankle’ App as compared to the usual practice of providing injured athletes with written materials. In addition, as a secondary outcome measure, the cost-effectiveness will be assessed against usual practice.

**Methods/Design:**

The proposed study will be a randomised controlled trial. After stratification for medical caregiver, athletes will be randomised to two study groups. One group will receive a standardized eight-week proprioceptive training program that has proven to be cost-effective to prevent recurrent ankle injuries, consisting of a balance board (machU/ MSG Europe BVBA), and a traditional instructional booklet. The other group will receive the same exercise program and balance board. However, for this group the instructional booklet is exchanged by the interactive ‘Strengthen your ankle’ App.

**Discussion:**

This trial is the first randomized controlled trial to study the implementation effectiveness of an App for proprioceptive balance board training program in comparison to a traditional printed instruction booklet, with the recurrence of ankle sprains among athletes as study outcome. Results of this study could possibly lead to changes in practical guidelines on the treatment of ankle sprains and in the use of mobile applications for injury prevention. Results will become available in 2014.

**Trial registration:**

The Netherlands National Trial Register NTR4027. The NTR is part of the WHO Primary Registries.

## Background

Ankle sprains are the most common sports and physical activity (PA) related injury [1-3]. It has been estimated that approximately 25% of all injuries across all sports are ankle injuries. Of all ankle injuries 85% involve the lateral ankle ligaments, i.e. acute lateral ankle sprains [3]. In the Netherlands, the most recent count of sports injuries showed that there is an estimated absolute number of 3.7 M acute sports injuries each year in a sporting population of 11 M athletes [4]. Of all annual sports injuries, approximately 530,000 are ankle sprains, of which almost 40 per cent requires (para) medical treatment [5]. Our research group has previously shown in a cost-effectiveness study [6] that, disregarding the requirement of medical treatment, the mean total (direct and indirect) cost of one ankle sprain is approximately €360. This would give a rough estimate of the annual sports-related ankle sprain costs in the Netherlands of €190,800,000. In addition, there is extensive evidence that there is an up to twofold-increased risk for ankle re-injury during the first year post-injury [6,7]. In fifty percent of all cases recurrences may result in disability and can lead to chronic pain or instability, requiring prolonged medical care [8]. As such, ankle sprains continue to pose a significant burden to the individual athlete, as well as to society as a whole.

Research has shown that both externally applied supports (i.e. taping or bracing of the ankle), as well as neuromuscular training programs are very successful in preventing recurrent ankle sprains, both from effectiveness, as a cost-effectiveness perspective [3,9-11]. While such measures have not been clearly linked to a primary preventive effect, the increased risk of recurrent injury can be reduced to the same level as previously uninjured athletes.

Therefore, in all current ruling treatment guidelines secondary preventive measures - preferably through continued neuromuscular training - are recommended after rehabilitation. These secondary preventive efforts regarding ankle sprains have been associated with high short-term returns on investment. The neuromuscular program that will be the centre of the proposed project has been linked to a €100 net return for each intervention package distributed [10].

However, despite ankle sprains being the single most common sports injury and despite an active approach by various Dutch organizations in implementing effective preventive measures and interventions, large-scale community uptake of preventive measures, and thus actual prevention of ankle sprains, is lagging well behind. This challenge can be derived from the Dutch injury rates registered by the Dutch Consumer Safety Institute VeiligheidNL [5], indicating that ankle sprain rates, treated at hospitals’ Emergency Departments, are consistent over the past years. In addition, the previously mentioned neuromuscular training program, that has been proven effective [8] and cost-beneficial [6], has been shown to have poor compliance [12]. In fact, the preventive effect in former studies was achieved in a subsample of compliant athletes, nevertheless showing significant population effects. Although analyses have been done from an intention-to-treat approach, this shows there is a lot to gain at an individual as well as a population level by increasing compliance to these simple and effective measures that are being advocated in various treatment guidelines.

In an attempt to bridge this implementation gap, VeiligheidNL looked into the possible role of new (social) media and has developed a freely available interactive ‘Strengthen your ankle’ App; available for iOS and Android) that contains - next to general advice on bracing and taping - the cost-effective neuromuscular program, as evaluated in a previous trial. This App provides the user withvideos and an interactive neuromuscular exercise schedule. It is a general belief that such interactive, online and mobile methods of information transfer are the way forward in prevention and implementation efforts. However, this has not yet been formally established for the uptake of injury preventive measures, and - although user reviews are positive - the ‘Strengthen your ankle’ App has not been evaluated against the well-studied ‘regular’ approach to advocate the neuromuscular program through written materials. Furthermore, if the ‘Strengthen your ankle’ App indeed does increase intervention uptake this will provide the necessary validation to further develop and enhance this promising role of new media in the implementation of preventive measures and interventions.

### Objectives

The objective of this randomised controlled trial is to evaluate the implementation value of the ‘Strengthen your ankle’ App as compared to the usual common practice of providing injured athletes with written materials.

Our hypothesis is that the use of the ‘Strengthen your ankle’ App will increase compliance to the prescribed neuromuscular training program and, consequently, will decrease ankle sprain recurrence incidence.

Specific research questions that will be answered are:

• What is the compliance to the prescribed 8-week exercise program via the App and via written material?

• Is there a difference in program compliance rates between the ‘Strengthen your ankle’ App and written materials?

• Is there a difference in ankle sprain recurrence incidence rates during a 12-month follow-up, between groups applying the ‘Strengthen your ankle’ App and written materials?

• Is there a difference in direct and indirect costs during a 12-month follow-up, between groups applying the ‘Strengthen your ankle’ App and written materials?

• Is there a difference in ankle sprain residual complaints (i.e. instability, feeling of giving way, pain, and continued sports participation) after a 12-month follow-up, between groups applying the ‘Strengthen your ankle’ App and written materials?

• What is the participants’ user experience of the ‘Strengthen your ankle’ App and the written materials?

## Methods

### Design

**Figure 1:**
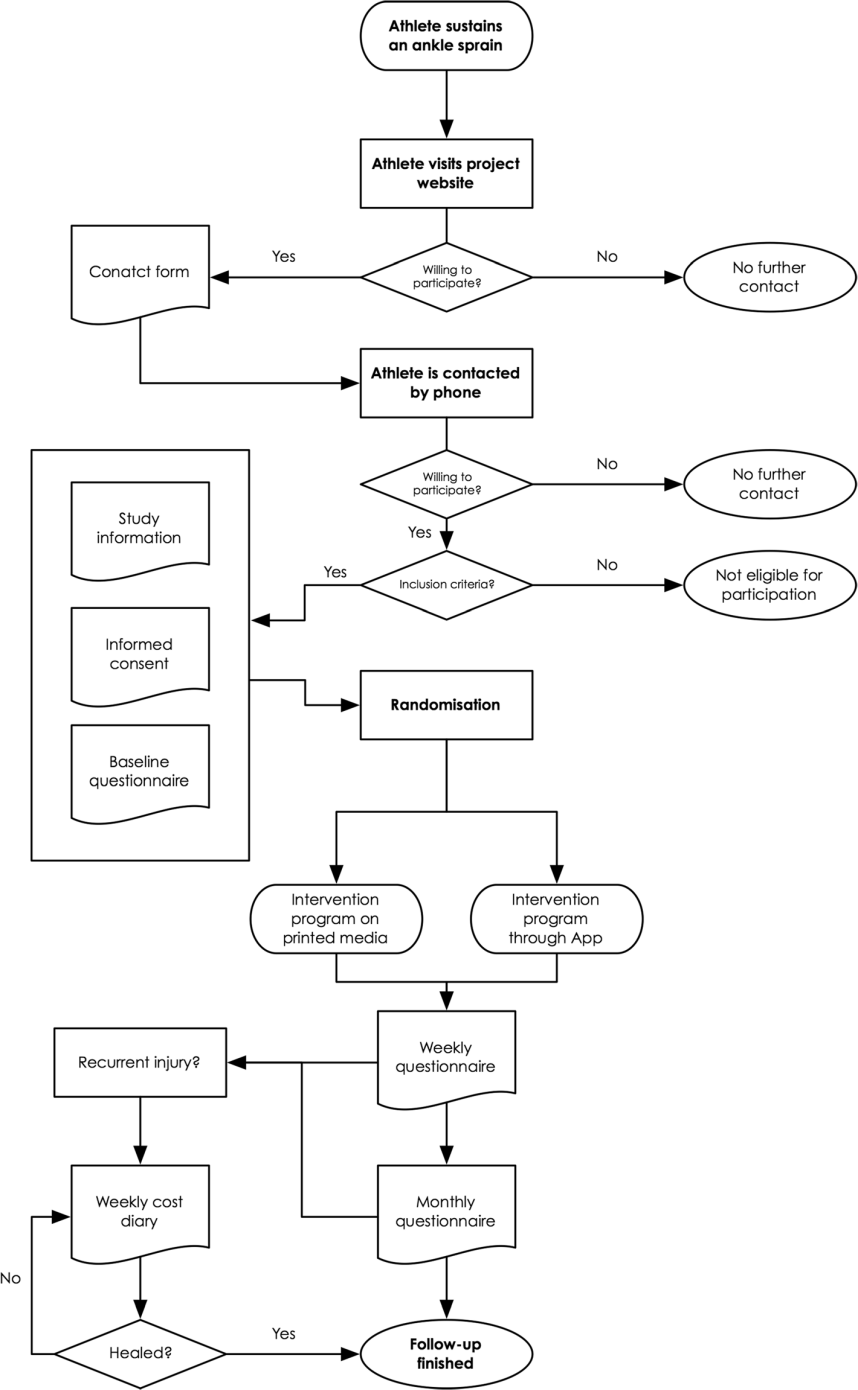
Study design and flow of the athletes.

The proposed study will be a randomised controlled trial. The study design and flow of the athletes are shown in Figure 1. The study design, procedures and informed consent procedure were approved by the Medical Ethics Committee (no. 2013/248) of the VU University Medical Center Amsterdam (VUmc), the Netherlands. The trial is registered in the Netherlands Trial Registry (NTR4027).

### Participants

Active participants (athletes), between 18 and 70 years of age, who have sustained an ankle sprain within the past two months, are eligible for inclusion. Responders are excluded if they have suffered from an injury different from a lateral ankle sprain in the same ankle (e.g. fracture of the ankle) in the previous year. Athletes should own a mobile phone with either Android of iOS. Athletes will be recruited through participating caregiving practices, websites from national sport federations, newsletters, an open invitation via the Internet and through the communication channels of participating sport associations.

### Study outline

#### ***Randomisation procedure***

After athletes have finished ankle sprain treatment by means of usual care, they will be randomised to one of the two study groups with stratification for initial treatment (i.e. medical or non-medical). Randomisation will take place at the end of treatment. This will minimise the risk of allocation bias. In addition, this will provide room to contact the medical care provider(s) involved in the athletes’ treatment. Medical care providers will be informed about the study in which the athlete partakes and will be asked to follow their usual treatment and/or rehabilitation program. Furthermore, they will be asked to encourage the athlete to take up their allocated intervention program after treatment and/or rehabilitation has ceased.

Athletes allocated to the ‘regular’ intervention group will receive a standardized eight-week proprioceptive training program, consisting of a balance board (machU/ MSG Europe BVBA), and an instructional booklet. This program has been shown to be effective in reducing recurrence injury risk in previous randomized controlled studies [9,10].

**Figure 2:**
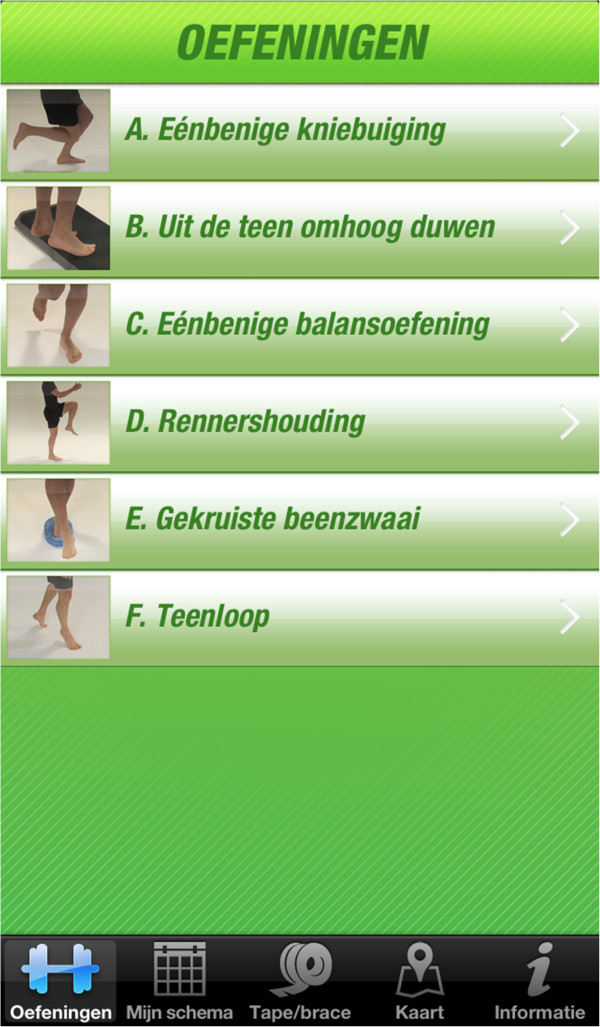
Basic exercises of the ‘Strengthen your ankle’ proprioceptive training program.

Athletes allocated to the ‘App’ group will also receive a balance board (machU/ MSG Europe BVBA), but the standardized eight-week proprioceptive training program will be provided through an interactive smartphone application, which is freely available for Android and iOS users. These two platforms are the most commonly used operating systems on smartphones (of all smartphones 79.3% runs on android, 13.2% on iOS) [13]. Thereby, selection bias is considered minimal. All athletes receive the same balance board. Both the instruction booklet and the ‘Strengthen your ankle’ App contain the same training program and six basic exercises (Figure 2).

### Baseline measurement

The online baseline questionnaire gathers information of each athlete on demographic variables, physical characteristics, sports & injury history, use of preventive measures, knowledge on injury prevention, severity of the current ankle sprain and subsequent treatment and/or rehabilitation.

### Follow-up measurement

After the 8-week training program, athletes will receive an online follow-up questionnaire to measure residual complaints of the initial ankle sprain and attitude towards the prescribed exercises. Both pain and feeling of giving way will be scored on five-point Likert scale for a series of questions.

Recurrent injury incidence and cost of injury outcomes will be measured once a month for a total period of 12 months. The follow-up measurements will gather information for each athlete on ankle sprains sustained during the preceding month, including details and mechanisms of this sprain and absence from sports due to the ankle sprain recurrence as a measure of recurrence severity. Finally, these online follow-up questionnaires will measure residual complaints of the initial ankle sprain. Both pain and feeling of giving way will be scored on five-point Likert scale for a series of questions, e.g. do you feel pain when being active, do you feel pain when getting out of bed in the morning, do you feel your ankle giving way when walking across the street, etc. At the last follow-up measurement (12 months) residual complaints of the initial ankle sprain will be measured again in all athletes.

### Compliance

Compliance (primary outcome) measurements will commence after randomisation (i.e. after treatment and at the start of the allocated intervention), and will take place weekly for the duration of the program (8 weeks). These measurements will gather information for each athlete on the number and sets of executed exercises. In addition, online questions will be asked regarding the clarity of the instructions provided, difficulty of the exercises and recurrence of an ankle sprain.

### Cost diary

In order to evaluate the cost-effectiveness of the allocated interventions, athletes who sustain an ankle sprain recurrence will be contacted by phone to obtain information on costs associated with treatment. Based on this information direct and indirect costs resulting from the sustained ankle sprain recurrence will be calculated for use in an economic evaluation. The economic evaluation will be performed from a societal perspective.

### Cost-effectiveness evaluation

Costs of the allocated intervention will include costs that are directly related to the implementation of the allocated intervention program. These costs include the written information materials, the development and maintenance of the application, and the balance boards. In addition to the cost of the intervention itself, direct health care costs will be included, i.e. costs of care by a general practitioner, physiotherapist, massage therapist, alternative therapist, sports physician or medical specialist (e.g., orthopaedic surgeon, general surgeon); hospital care, use of drugs (e.g. acetaminophen, ibuprofen) and the use of medical devices (e.g., crutches, tape, braces). The costs of drugs will be estimated on the basis of prices recommended by the Royal Dutch Society of Pharmacy [14]. Also, indirect costs resulting from a loss of production due to absenteeism from paid or unpaid work will be included. Indirect costs for absenteeism from paid work are calculated using the friction cost approach of 4 months, based on the mean age and sex specific income of the Dutch population. Indirect costs for productivity loss of unpaid work, such as study and household work, costs are estimated at a shadow price of €8.30 an hour [15].

### Sample size

Sample size calculations are based upon the primary outcome measure compliance, and are based upon previously established compliance rates to the same program when advocated through written materials [12]. Full compliance rates in the written materials’ group are expected to be around 25%. A doubling of this rate to at least 50% is considered to be clinically relevant. Based upon a beta of 0.90 and an alpha of 0.05 a total of 158 athletes is required divided across both study groups. In our experience from previous comparable studies the dropout rate during a 12 months follow-up is about 20%. This would mean that a sample of 190 athletes is needed per group.

### Recruitment of study population

Physical therapy and physician practices will aid in the recruitment of athletes. Participating practices will be instructed on the aim, background and procedures of the study. Athletes treated for an ankle sprain at participating practices will be informed of the study by their caregiver. Athletes willing to participate, will then be contacted by the research team by phone after which they will enrol in the study.

Athletes will also be recruited through the Internet. Calls will be placed on the websites of associations of sports with a relatively high ankle sprain rate (volleyball, handball, basketball, korfball, soccer and athletics), websites of organisations participating in this study and on sports-related websites (e.g. http://www.meetingpoint.nl, http://www.runinfo.nl, etc.). Where possible, existing mailing lists of sport associations will be used to contact potential athletes directly. In addition electronic newsletters will be used for active recruitment of athletes.

The same recruitment strategy as described above has been employed successfully in two previous studies on the same topic [16,17]. In both studies a larger sample of injured athletes was successfully included, 476 and 352 athletes respectively.

One of the drawbacks of this method of inclusion is that we have no control over the treatment that is being given or has been given for the current ankle sprain. Although ruling guidelines are considered usual care, this does not necessarily mean that caregivers are actually following these guidelines by the book. Inclusion of athletes through a limited number of controlled (para) medical caregivers would decrease this problem. However, as we have learned in previous studies, inclusion through such channels is problematic and almost always results in lower inclusion rates than expected. Even so, in the proposed study we are looking for athletes treated by a variety of (para) medical caregivers. Meaning that in the proposed study a relatively large number of different caregivers would need to be found, informed on the study, and controlled as to their given treatment. Looking at the required number of athletes we believe this would prove an undoable and unrealistic undertaking. Moreover, the proposed study is on the effect of secondary preventive measures that are being applied after treatment by the (para) medical caregiver. When the caregiver would perform inclusion, this means that randomisation needs to take place at the level of the caregiver. This further complicates the study design.

### Usual care as employed in the current study

For the current study, usual care is defined as any care the athlete might seek or receive after an ankle sprain. We also define self-treatment to be usual care in the current study. Next to treatment by a (para) medical professional 60 per cent of ankle sprains - mostly minor- is self-treated by the athlete [5]. Consequently these athletes do not receive the care as described in the below mentioned ruling guidelines.

In case the athlete does receive (para) medical care, there are two ruling medical guidelines for the treatment of ankle sprains in the Netherlands, i.e. the Royal Dutch Physiotherapy Association (KNGF) guideline [18] and the Dutch Institute for Healthcare Improvement (CBO) guideline [19]. The KNGF guideline, which is the most commonly employed, aims at optimal functional recovery of the ankle, returning to full sports participation and preventing recurrent ankle injuries. Rehabilitation consists of three phases: phase 1 which aims to reduce pain and swelling, phase 2 in which load is gradually increased and functionality is re-established and phase 3 in which normal average daily living (ADL) tasks are performed. After full rehabilitation athletes are advised to use secondary preventive measures. Whereas elite athletes could have treatment duration of up to twelve weeks, six weeks are considered sufficient for amateur athletes, according to the KNGF guidelines [18].

For the purpose of the current study we do not interfere in the athletes choice of caregiver and the caregivers’ compliance to the ruling guidelines.

### Statistical analyses

All analyses will be carried out according to the intention-to-treat principle.

Compliance rates between groups will be compared by means of a multivariate linear regression analysis using compliance as a continuous dependent variable. Cox-regression analysis will be used to compare ankle recurrence risk between the intervention and the control group. Absence from sports will be compared between the two groups using a Mann–Whitney test, since absence from sports due to an injury is not normally distributed. For all analyses, variables will be checked for confounding and/or effect-modification and will be adjusted for accordingly.

Mean direct, mean indirect and total costs will be estimated and compared between the two groups, both for the costs per athlete in the injured population and for the costs per athlete in the total population. Because costs will not be normally distributed, 95% confidence intervals for the differences in mean costs will be obtained by bias-corrected and accelerated bootstrapping with 2000 replications. Differences in costs and differences in ankle sprain recurrences will be included in a cost-effectiveness ratio, which estimates the incremental costs to prevent one ankle sprain recurrence. Confidence intervals for the cost-effectiveness ratio will be calculated with bootstrapping, using the bias-corrected percentile method with 5000 replications. Uncertainty of this ratio will be evaluated by presenting a cost-effectiveness plane and sensitivity analyses will be performed to check the robustness of the results. An acceptability curve will also be presented.

### Discussion

The results of this study can possibly lead to a change in the treatment of ankle sprains. Positive results can offer extended possibilities for implementation of the intervention in usual care. Positive study results can also lead to changes in the practical guidelines on the treatment of ankle sprains. Furthermore, if the ‘Strengthen your ankle’ App indeed does increase intervention uptake this will provide the necessary validation to further develop and enhance this promising role of new media in the implementation of preventive measures and interventions.

## Competing interests

The authors declare no competing interest.

## Authors’ contributions

EV (e.verhagen@vumc.nl) conceived the research idea. MVR (m.vanreijen@vumc.nl) and EV have written the protocol. MVR will screen and include patients, perform data analysis and be the main author of articles on the primary aim of the study. IV (i.vriend@veiligheid.nl), WVM (w.vanmechelen@vumc.nl) and VZ (v.zuidema@vumc.nl) contributed to ideas in the protocol. All authors have read and commented on the draft version and approved the final version of the manuscript.

## Pre-publication history

The pre-publication history for this paper can be accessed here:

http://www.biomedcentral.com/1471-2474/15/2/prepub
